# Supplementary material for: stilPCR increases the effective sequencing length of Illumina targeted next-generation sequencing
Source: PLoS One. 2024 Dec 9;19(12):e0314265. doi: 10.1371/journal.pone.0314265 (PMC11627403; doi:10.1371/journal.pone.0314265)
Supplement: S1 File — (PDF) [file pone.0314265.s001.pdf]

Nov 11, 2024

stilPCR (single-tube Illumina long read PCR) increases the effective sequencing length of Illumina, targeted next-generation sequencing

DOI
dx.doi.org/10.17504/protocols.io.bp2l6xmn5lqe/v1

Jason D Limberis[1], Roland Nagel[2], Soumitesh Chakravorty[2], Dena Block[2], Scott Dewell[2], Alina Nalyvayko[1], Zach Howard[1], Grant Theron[1], Rouxjeane Venter[1], John Metcalfe[1]

[1]University of California, San Francisco; [2]Cepheid Inc

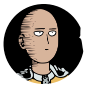

Jason D Limberis
University of California, San Francisco

OPEN ACCESS

DOI: dx.doi.org/10.17504/protocols.io.bp2l6xmn5lqe/v1

Protocol Citation: Jason D Limberis, Roland Nagel, Soumitesh Chakravorty, Dena Block, Scott Dewell, Alina Nalyvayko, Zach Howard, Grant Theron, Rouxjeane Venter, John Metcalfe 2024. stilPCR (single-tube Illumina long read PCR) increases the effective sequencing length of Illumina, targeted next-generation sequencing. **protocols.io https://dx.doi.org/10.17504/protocols.io.bp2l6xmn5lqe/v1**

License: This is an open access protocol distributed under the terms of the **Creative Commons Attribution License**, which permits unrestricted use, distribution, and reproduction in any medium, provided the original author and source are credited

Protocol status: Working
We use this protocol and it's working

Created: November 06, 2023

Last Modified: November 11, 2024

Protocol Integer ID: 90500

Keywords: tuberculosis, illumina, NGS, sequecing

Funders Acknowledgement:
National Institute of Allergy and Infectious Diseases
Grant ID: R01AI177637

Abstract

Identifying pathogens, resistance-conferring mutations, and strain types through targeted amplicon sequencing is an important tool. However, due to the limitations of short read sequencing, many applications require the division of limited clinical samples. Here, we present stilPCR (single-tube Illumina long read PCR), which allows the generation of hemi-nested amplicons in a single tube, with Illumina indexes and adapters, effectively increasing the Illumina read length without increasing the input requirements of reagents or sample. We have successfully utilized stilPCR on clinical sputum from tuberculosis patients to detect drug resistance mutations.

Materials

Required

Q5 Hot Start High-Fidelity DNA Polymerase - 500 units **New England Biolabs Catalog #**M0493L

Agencourt AmPure XP beads **Contributed by users Catalog #**A63880

dNTPs **Contributed by users**

A thermocycler and a qPCR machine

A magnetic rack

Optional

NEBNext Library Quant Kit for Illumina - 500 rxns **New England Biolabs Catalog #**E7630L

A	B	C
Primer Set	Direction	Sequence
Rv0678_F1	F	ACACTCTTTCCCTACACGACGCTCTTCCGATCTAGTTCCAATCATCGCCCTCCGC
Rv0678_F2	F	ACACTCTTTCCCTACACGACGCTCTTCCGATCTTGACTCGGTTGGCGGGTCGA
Rv0678_R	R	GACTGGAGTTCAGACGTGTGCTCTTCCGATCTGCCGTCTTGCTCGCCACCTC
TruSeq_UT_i5_F	F	AATGATACGGCGACCACCGAGATCTACACTG[i5]ACTCTTTCCCTACACGACGCTCTTCCGATCT
TruSeq_UT_i7_R	R	CAAGCAGAAGACGGCATACGAGAT[i7]GTGACTGGAGTTCAGACGTGTGCTCTTCCGATCT

Primer sequences used in this study.

Stage 1 PCR

1

A	B	C
COMPONENT	Conc.	50ul
Reaction Buffer	5X	10
GC Buffer	5X	10
10 mM dNTPs	10 mM	1
Q5 High-Fidelity DNA Polymerase	2000 units/ml	0.5
Rv0678_F1	10 µM	1
Rv0678_R	10 µM	0.5
TruSeq_UT_A702_R	10 µM	0.5
BSA	20 mg/ml	5
Template DNA	variable	5
Nuclease-Free Water	NA	17

The total volume is 50ul at this stage

A	B	C	D
Step	Temp (C)	Time (s)	Cycles
Denaturation	98	120	1
Denaturation	98	10	4
Annealing	64	20	
Extension	72	120	
Denaturation	98	10	18
Annealing	65	15	
Extension	72	60	
Extension	4	Forever	1

Cycle parameters

As soon as the sample hits 4 °C , proceed to step 2.

Stage 2 PCR

2 Add in the below to the same reaction tube.

A	B	C
COMPONENT	Conc.	50ul
Reaction Buffer	5X	1
Rv0678_F2	10 µM	0.5
TruSeq_UT_A503_F	10 µM	1.5
TruSeq_UT_A702_R	10 µM	1.5

The total volume is 55ul at this stage

A	B	C	D
Step	Temp (C)	Time (s)	Cycles
Denaturation	98	10	1
Denaturation	98	10	12
Annealing	65	15	
Extension	72	60	
Extension	72	120	1

Cycle parameters

Bead cleanup

3 Add 25 µL (1X) of resuspended AMPure XP Beads to the sample
Mix by pipetting 10x

4 Incubate 00:02:00 at Room temperature

5 Place on the magnet, allow the beads to aggregate, and remove and discard the supernatant

6 Add 200 µL [M] 70 % (v/v) ethanol and incubate (still on the magnet) for 00:00:30

6.1 Remove the supernatant

6.2 Repeat for a total of 2 washes

7 Air dry for 00:00:30 , don't allow the beads to become cracked

8 Immediately after the bead pellet becomes opaque, remove the tube from magnetic rack and resuspend in 20 µL of **Low EDTA Tris Buffer**. Ensure all beads are in solution.

9 Incubate at room temperature for 00:05:00

10 Place on magnetic rack, wait for the solution to become clear ~ 00:02:00 , and transfer the eluted DNA to a new well-labeled tube

Optional: NEB Illumina Quantification

11 Thaw the NEBNext Library Quant Master Mix and NEBNext Library Quant Primer Mix. Ensure mixing of NEBNext Library Quant Primer Mix by vortexing. Place reagents on ice.

12 Thaw the NEBNext Library Quant DNA Standards, tubes 1–6.
Mix by pulse vortexing on a low setting. Briefly spin to collect material from the sides of the tubes. Place on ice.

13 Thaw the NEBNext Library Quant Dilution Buffer (10X). Mix well by vortexing.
Centrifuge briefly to collect material from the sides of the tube.
Place on ice.

14 Add 100 µL NEBNext Library Quant Primer Mix to the tube of NEBNext Library Quant Master Mix (1.5 mL). Mix by vortexing. Write the date on the master mix tube to indicate that primer mix has been added.

15 Dilute the NEBNext Library Quant Dilution Buffer (10X) 1:10 with nuclease-free water. Mix by vortexing.
Prepare sufficient buffer for quantitating the desired number of libraries, allowing 1.2 mL for each library.

16 Prepare a 1:1,000 dilution of each library sample in NEBNext Library Quant Dilution Buffer (1X)

17 Aliquot 16 µL NEBNext Library Quant Master Mix (with primers) to each well

18 Add-in 4 µL of sample or standard per well

19

A	B	C	D
Step	Temp (C)	Time (s)	Cycles
Denaturation	95	60	1
Denaturation	95	15	35
Annealing	63	45	

Cycle parameters

A denaturation/melt curve can be included if desired, but is optional.

A	B
Sample	Conc. (pM)
DNA Standard 1	100
DNA Standard 2	10
DNA Standard 3	1
DNA Standard 4	0.1
DNA Standard 5	0.01
DNA Standard 6	0.001

20 Adjusted Conc. = Calculated Conc. × 399 / library size (bp)
For library size, use the average of the two amplicon sizes.

Data processing

21 Data processing can be done using the stilPCR pipelineavailable here (**link**).

```
bash stilPCR.sh \
  --R1 "test_data/read_R1_001.fastq.gz" \
  --R2 "test_data/read_R2_001.fastq.gz" \
  --ref "refs/BDQ_duplex.fasta" \
  --primers "refs/primers.bed"
```

Expected Outcome

22

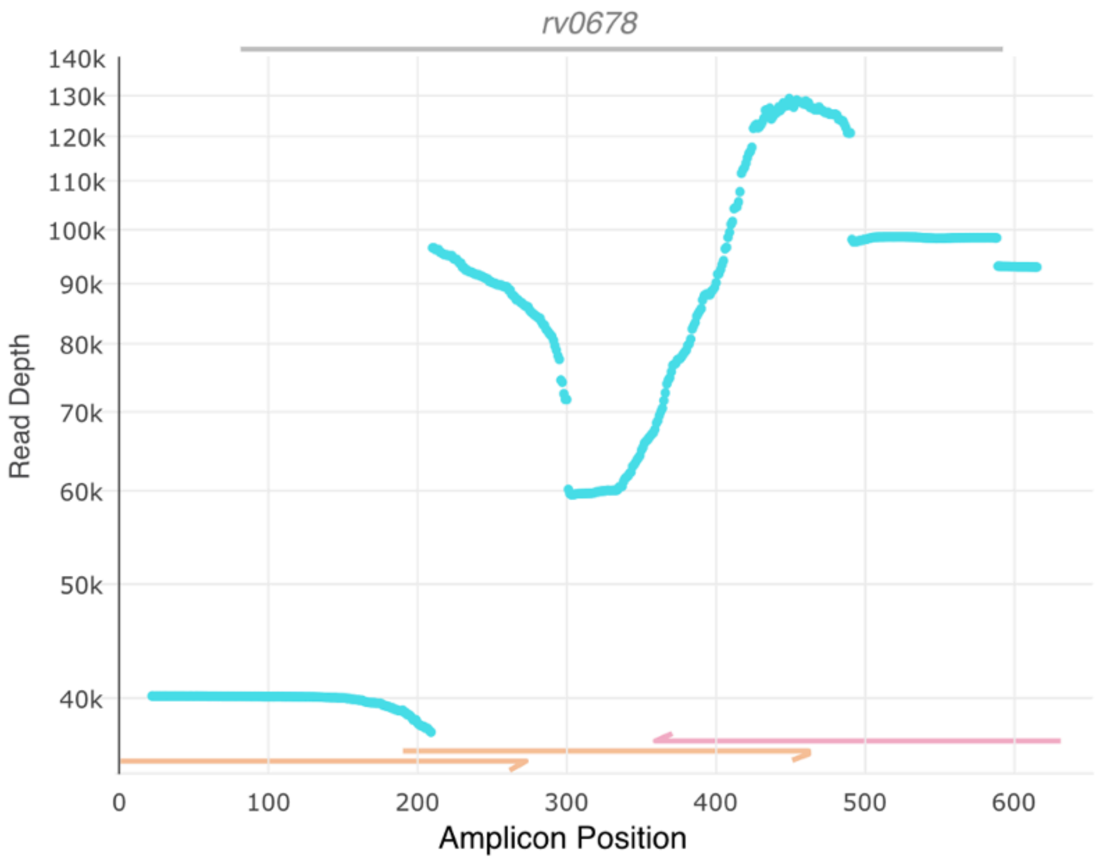

Successful sequencing of *rv0678* and its promoter region using stilPCR. The plot shows the coverage (y-axis) at each position (x-axis) along the sequence amplicons. The coding region is denoted by the grey line at the top of the figure. The positions of forward primer sequencing amplicons are shown in orange at the bottom of each plot, with reverse primer amplicons in pink.
