# Supplementary material for: stilPCR increases the effective sequencing length of Illumina targeted next-generation sequencing
Source: PLoS One. 2024 Dec 9;19(12):e0314265. doi: 10.1371/journal.pone.0314265 (PMC11627403; doi:10.1371/journal.pone.0314265)
Supplement: Attachment — Submitted filename: reviewr_responses_R1.docx [file pone.0314265.s002.docx]

PONE-D-24-37091

stilPCR increases the effective sequencing length of Illumina, targeted next-generation sequencing.

PLOS ONE

Thank you for the opportunity to submit a revised protocol and we thank the reviewers for their comments and suggestions, answering them has strengthened the manuscript. We have responded to the specific concerns below (in blue text) and made the relevant changes to the manuscript and protocol. Thank you for your consideration, and we look forward to hearing from you.

Reviewers' comments:

Reviewer's Responses to Questions

**Comments to the Author**


1. Does the manuscript report a protocol which is of utility to the research community and adds value to the published literature?

Reviewer #1: Yes

Reviewer #2: Yes

2. Has the protocol been described in sufficient detail?


To answer this question, please click the link to protocols.io in the Materials and Methods section of the manuscript (if a link has been provided) or consult the step-by-step protocol in the Supporting Information files.


The step-by-step protocol should contain sufficient detail for another researcher to be able to reproduce all experiments and analyses.

Reviewer #1: Partly

Reviewer #2: Partly

3. Does the protocol describe a validated method?

The manuscript must demonstrate that the protocol achieves its intended purpose: either by containing appropriate validation data, or referencing at least one original research article in which the protocol was used to generate data.

Reviewer #1: Yes

Reviewer #2: Yes

4. If the manuscript contains new data, have the authors made this data fully available?

The [PLOS Data policy](http://www.plosone.org/static/policies.action#sharing) requires authors to make all data underlying the findings described in their manuscript fully available without restriction, with rare exception (please refer to the Data Availability Statement in the manuscript PDF file). The data should be provided as part of the manuscript or its supporting information, or deposited to a public repository. For example, in addition to summary statistics, the data points behind means, medians and variance measures should be available. If there are restrictions on publicly sharing data—e.g. participant privacy or use of data from a third party—those must be specified.

Reviewer #1: Yes

Reviewer #2: Yes

**5. Is the article presented in an intelligible fashion and written in standard English?**


PLOS ONE does not copyedit accepted manuscripts, so the language in submitted articles must be clear, correct, and unambiguous. Any typographical or grammatical errors should be corrected at revision, so please highlight any specific errors that need correcting in the box below.

Reviewer #1: Yes

Reviewer #2: Yes

6. Review Comments to the Author

Please use the space provided to explain your answers to the questions above. You may also include additional comments for the author, including concerns about dual publication, research ethics, or publication ethics. (Please upload your review as an attachment if it exceeds 20,000 characters)

Reviewer #1: Overall this is a clever method for increasing the length of short read amplicon sequencing, while sustaining current sample input and sequencing reagent requirements. Although the authors focus on conferring mutations in pathogens relevant to human health, the described technique could be broadly applied to amplicon sequencing strategies requiring additional length beyond the traditional methods.

Recommendations for revision:
-The intro states the short read limitation of Illumina sequencing as 500 bp or 250x2 PE reads. Illumina offers 600 bp/ 300x2 PE read kits for MiSeq and NextSeq 1k/2k.

Thanks, we have updated this in the introduction.

-It would be helpful to know the total bp size required for the current technique used to confer resistance mutations in TB.

We have clarified this in the expected results. “We have successfully used stilPCR to amplify two targets, *atpE, and rv0678,* of *Mycobacterium tuberculosis* in patient sputum[1]. *rv0678* is a 498bp gene and mutations along its entirety may confer *M. tuberculosis* bedaquiline resistance[2,3].”

-The provided GitHub link, www.github.com/SemiQuant/stilPCR, leads to a 404 error.
-"Controlling the concentrations of the gene-specific primers allows us to produce the
different amplicons at similar concentrations" How was this validated? Determined by the number of reads per primer, qPCR?

Sorry, we had the GitHub repository set to private, we have now made it public. We have also added the following to claify our statement regarding the primer concentrations.

“The tail sequences allow for even amplification of tailed products of different sizes. Controlling the concentrations of the gene-specific primers will allow you to produce the different amplicons at similar concentrations. The specific concentration of primer required can be determined by adding 0.5ul of 2000X SYBR green dye to the PCR reaction and monitoring the Ct values of the single plex reactions using a qPCR thermocycler. You can also control the maximum number of amplicons for Illumina cluster generation (sequencing) and ensure the maximum amount of generated data per sample by limiting our universal-tailed primers. Importantly, stilPCR-generated libraries can be pooled with any standard Illumina library, allowing for batching even with other sample types.”

-Are the primer sequences used available? I was unable to find them in the manuscript, Protocols.IO page, or SI. Please include them or where to find them.

Apologies, we have added them to the materials section of the protocol.

Reviewer #2: In this work, the authors describe a targeted amplicon sequencing protocol that generates both full-length and partial-length amplicons spanning a chosen genomic location. This is accomplished using hemi-nested PCR and results in the ability to sequence across genomic locations longer than would be possible using paired-end short read sequencing on an Illumina platform. The authors demonstrate the utility of the protocol on patient sputum by amplifying, sequencing, and mapping reads across a target on the Mycobacterium tuberculosis genome used for identifying resistance to a particular antibiotic. This method is very useful for analyses on samples for which there exists a very limited quantity since template for only one reaction tube is needed to amplify the longer genomic regions. In addition, although long read sequencing can be accomplished using other sequencing technologies, employing Illumina sequencing technology is important for variant calling because of the higher sequence quality obtained. The technique is well-explained, and the manuscript is well-written, however a few minor edits will bring valuable clarity for readers.

1. In the Expected Results section, only one example is depicted. While the figure clearly demonstrates the results, including only one example does not give information for readers to assess the reproducibility of the technique. Inclusion of another example figure along with a sentence regarding reproducibility would be helpful.

We have added the following to the expected results section: “Further examples demonstrating the reproducibility of stilPCR are available in Limberis et al[1].”

2. In the Protocol, the first table in Stage 1 PCR does not list concentrations. Concentrations of the primers and BSA should be included.

We have added all the concentrations of the reagents used.

3. In the Protocol, the total volume for Stage 2 PCR is given as 55 uL. Adding the additional 4.5 uL listed would result in a total volume of 54.5 uL. Is the table missing a component or is the 55 uL obtained by rounding up?

Apologies, we have corrected the volume of water in the first step, it was 16.5ul but should be 17ul.

7. PLOS authors have the option to publish the peer review history of their article ([what does this mean?](https://journals.plos.org/plosone/s/editorial-and-peer-review-process#loc-peer-review-history)). If published, this will include your full peer review and any attached files.


If you choose “no”, your identity will remain anonymous but your review may still be made public.


**Do you want your identity to be public for this peer review?** For information about this choice, including consent withdrawal, please see our [Privacy Policy](https://www.plos.org/privacy-policy).

Reviewer #1: No

Reviewer #2: No
